# Complete mitochondrial genome of the diving beetle, *Cybister brevis* Aubé, 1838 (Coleoptera, Dytiscidae) from Jeju Island

**DOI:** 10.1080/23802359.2024.2317327

**Published:** 2024-02-22

**Authors:** Dae-Ju Oh, Min-Hee Ko, Dong-Won Min, Yong-Hwan Jung

**Affiliations:** a Biodiversity Research Institute, Jeju Technopark, Seogwipo, Jeju, Republic of Korea; b The Bugs, Jeju, Republic of Korea

**Keywords:** *Cybister*, mitochondrial genome, phylogeny, Dytiscidae, evolutionary history

## Abstract

The mitochondrial genome of *Cybister brevis* Aubé, 1838, is 16,198 bp long and includes 37 genes that are highly conserved in the family Dytiscidae. Phylogenetic analysis revealed that all genera in Dytiscidae, except *Hydroporus* and *Oreodytes*, are monophyletic. The Hydroporini tribe was found to be polyphyletic and closely associated with the Hygrotini, Bidessini, and Hyphydrini tribes. Dytiscinae was found to be a highly divergent polyphyletic group comprising three distinct clades. This study also revealed that *C. brevis* is closely related to *Cybister japonicus* and that the tribe Cybistrini diverged relatively early compared to other tribes in Hydroporinae. These findings are consistent with those of previous studies and provide new insights into the phylogeny of the Dytiscidae family, which has previously shown discrepancies between morphological characteristics and molecular data. The genome-level analyses conducted in this study serve as a valuable foundation for future investigations into the Dysticidae evolutionary history.

## Introduction

Dytiscidae, a family of predaceous diving beetles priorly estimated to contain approximately 5,000 species (Jäch and Balke 2008), was recently described by Nilsson and Hájek (2023) to contain 4,668 species, 97 of which belong in the genus *Cybister*.

**Figure 1.**
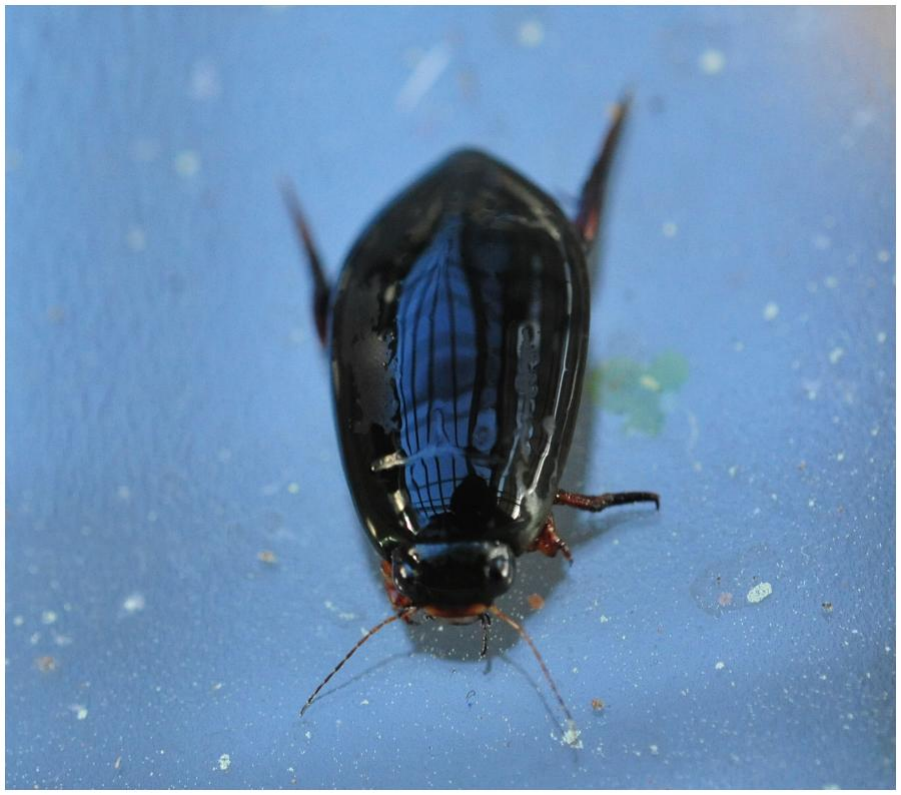
Photograph of *Cybister brevis*. This picture was taken by Min-Hee Ko.

The diving beetle, *Cybister brevis* Aubé, 1838 (Figure 1), is a predatory aquatic insect that belongs to the Dytiscidae family. While aquatic insects are less extensively studied than terrestrial insects, several studies have examined the phylogenetic relationships among aquatic insects, including Dytiscidae members, using molecular and morphological analyses (Shull et al. 2001; Miller et al. 2006; Alarie and Michat 2007; Ribera et al. 2008; Michat et al. 2017). However, there is generally no consensus between molecular and morphological analyses of aquatic insects. Therefore, additional investigations are required to obtain an accurate understanding of the phylogenetic relationships among aquatic insects.

Molecular data are useful for studying the taxonomy, phylogeny, and evolution of insects. Mitochondrial (mt) sequences have been analyzed in various taxa to determine their population dynamics and phylogenetic relationships (Deuve et al. 2012; Cameron 2014; Kim et al. 2017; Linard et al. 2018).

Owing to its rapid evolutionary rate and ease of sequencing using modern technology, mitochondrial genome (mtgenome) sequencing represents a valuable tool for phylogenetic analysis. It allows for dense taxon sampling, improved estimates of molecular rates and nucleotide composition, and reliable inferences of evolutionary relationships based on the presence of conserved protein-coding genes and a haploid inheritance pattern (Timmermans et al. 2016).

Here, mtgenome analysis of *C. brevis* was performed to verify the phylogenetic relationships among species in the Dytiscidae family and enhance our understanding of the evolutionary history of aquatic insects.

## Materials and methods

We sequenced the mtgenome of *C. brevis* obtained from the Halla Eco-Forest (33°25′56″N, 126°35′51″E) in Jeju Island, Korea. A specimen was deposited at the Biodiversity Research Institute, Jeju Technopark (http://jbridb.jejutp.or.kr, Min-Hee Ko, vampire5942@jejutp.or.kr) under voucher number JBRI-INSCT-042.

Genomic DNA was extracted from leg muscles of *C. brevis* using a Nucleospin Tissue Kit (Macherey-Nagel, Germany) following the manufacturer’s protocol. Extracted DNA was stored in a deep freezer at the Jeju Biodiversity Research Institute. The entire mtgenome was amplified and sequenced using newly designed primers, including nested primers for primer-walking sequencing. All sequencing reactions were conducted *via* polymerase chain reaction (PCR) and Sanger sequencing at Bionics Co., Ltd., (Seoul, Korea).

The complete mitochondrial genome was annotated using MITOS (Bernt et al. 2013) and ORF Finder (https://www.ncbi.nlm.nih.gov/orffinder) by using the invertebrate mitochondrial code. A mtgenome map was generated using the SnapGene 5 software. The tRNA genes were identified using the web-based tRNAscan-SE software (Lowe and Eddy 1997) and ARWEN (Laslett and Canbäck 2008).

To determine the phylogenetic relationships among Dytiscidae species, 61 mtgenome sequences were obtained from the GenBank database (NCBI, National Institutes of Health, Bethesda, MD, USA). Thirteen protein-coding gene (PCG) sequences were aligned using Clustal X (Thompson et al. 1997) on default settings. We used Bayesian inference coupled with MrBayes 3.2 software (Ronquist et al. 2012) to construct a phylogenetic tree. *Hygrobia hermanni* (KT876898) was used as the outgroup taxon for the analysis.

## Results

**Figure 2.**
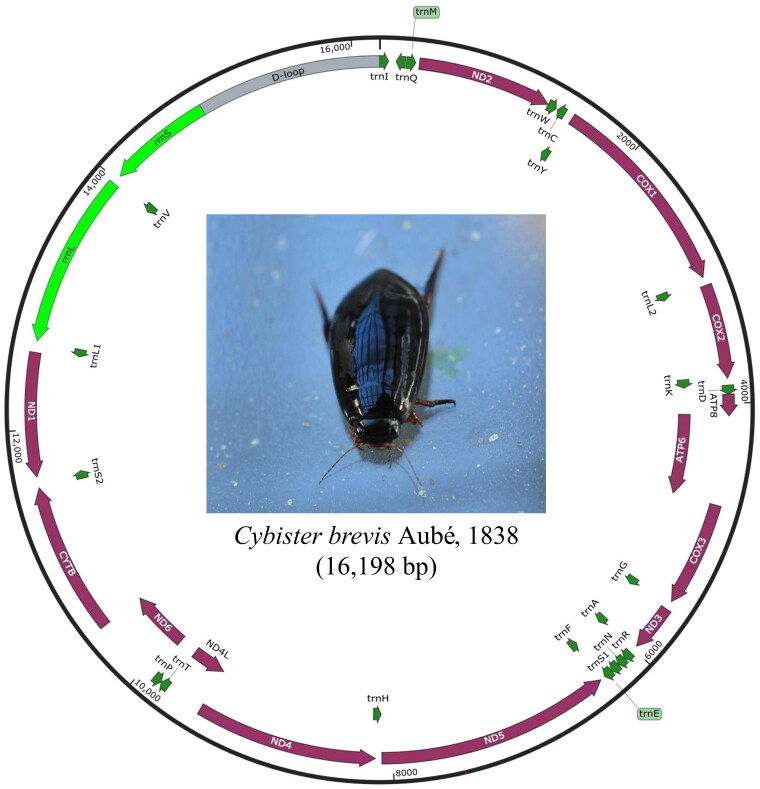
Circular map of the mitochondrial genome of *C. brevis*. Protein-coding genes, transfer RNA, ribosomal RNA, and control region are indicated using purple, dark green, light green, and gray colors, respectively.

The mtgenome of *C. brevis* was 16,198 bp in length (Figure 2) and the sequence was deposited in GenBank (accession no. MZ504983). The mtgenome contained 37 genes, including 13 PCGs, two rRNAs, and 22 tRNAs. Nine of the 13 mt PCGs were encoded on the heavy strand, whereas the NADH dehydrogenase subunit 1, 4, 4 L, and 5 genes were encoded on the light strand. Translation of all PCGs in the *C. brevis* mtgenome was initiated using ATT, ATG, and ATA and terminated by use of TAA and TAG. Fourteen of the 22 tRNAs (I, M, W, L2, K, D, G, A, R, N, S1, E, T, and S2) were encoded on the heavy strand, while eight tRNAs (Q, C, Y, F, H, P, L1, and V) were encoded on the light strand.

**Figure 3.**
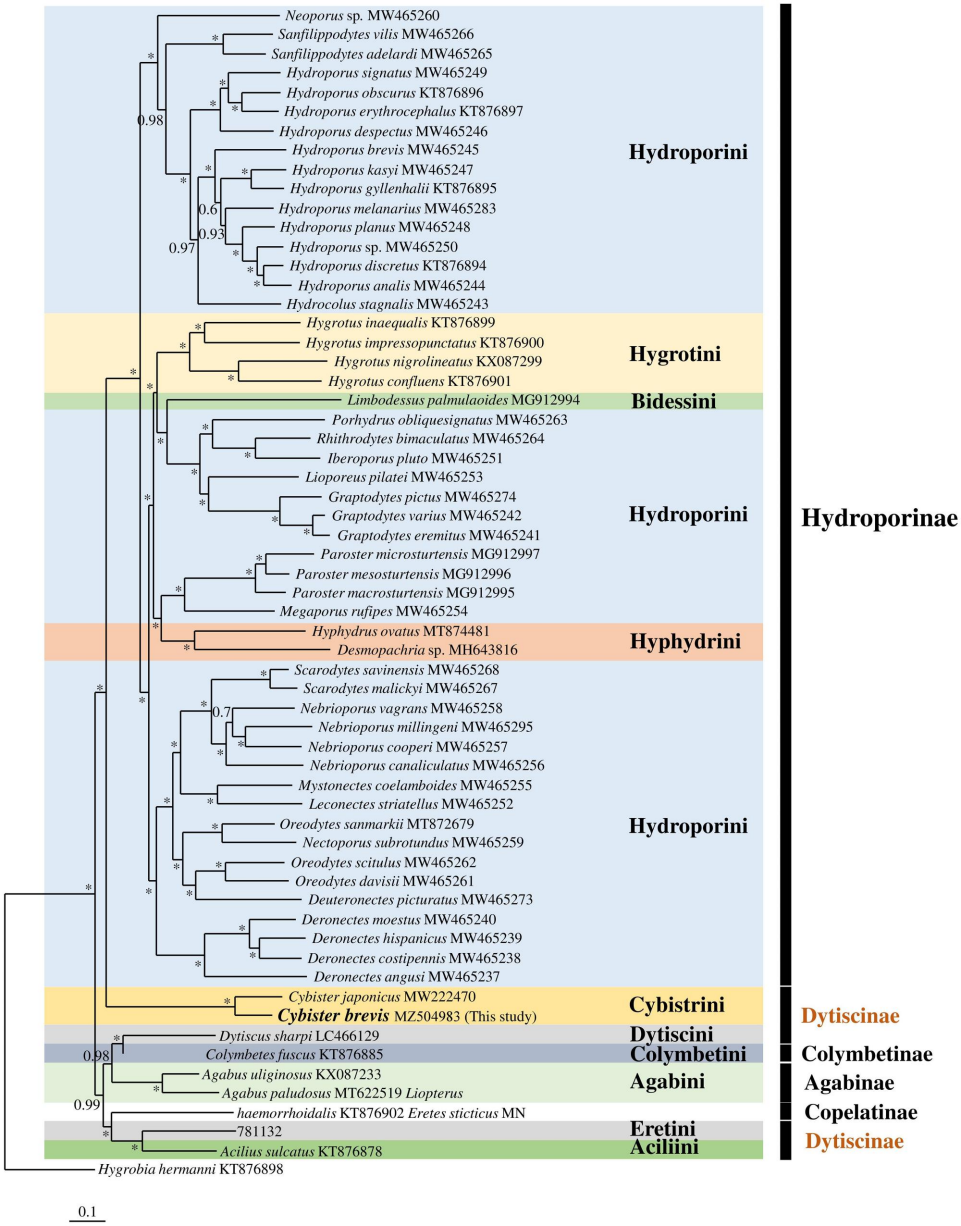
Molecular phylogenetic analysis of 13 protein-coding genes in the mitochondrial genome of 60 species of Dytiscidae. Four markov chains were run for 500,000 generations and sampled every 100 generations to yield a PP distribution (1.0 PP is indicated by an asterisk). *Hygrobia hermanni* was used as the outgroup taxon. PP, posterior probability.

Based on the phylogenetic analysis of Dytiscidae members (Figure 3), all genera were monophyletic, except *Hydroporus* and *Oreodytes. Hydroporus* formed a paraphyletic group with *Hydrocolus*, whereas *Oreodytes* was polyphyletic, as it formed a group with *Nectoporus* and *Deuteronectes*. Hydroporini was found to represent a polyphyletic group closely associated with the Hygrotini, Bidessini, and Hyphydrini tribes. Additionally, Dytiscinae was found to be a highly divergent polyphyly comprising three distinct clades.

## Discussion and conclusion

The mtgenome of *C. brevis* is structurally identical to that of other beetles (Hyde et al. 2018; Vasconcelos et al. 2021; Liu et al. 2023). The gene order of 13 PCGs, 22 tRNAs, and 2 rRNAs was highly conserved in Dytiscidae. Alternative initiation codons, including ATT, ATG, and ATA, have been reported in Dytiscidae mtgenomes (Hyde et al. 2018; Vasconcelos et al. 2021).

The phylogenetic tree indicated that *C. brevis* was closely related to *Cybister japonicus*, and the tribe Cybistrini formed a group with the tribes in Hydroporinae as a sister group (Figure 3). The tribe Cybistrini was found to have diverged relatively early compared to other tribes in Hydroporinae. This result was consistent with those of previous studies (Shull et al. 2001; Ribera et al. 2008) that analyzed phylogenetic relationships using four genes (*CO1*, *16S*, *H3*, and *12S*), suggesting that the branches of Cybistrini diverged before the divergence of branches of Hydroporinae. However, previous phylogenetic analyses based on morphological characteristics have suggested that Hydroporinae diverged before the Dytiscinae did (Michat and Torres 2009; Michat 2010).

Many previous studies have investigated the phylogenetic relationships among species within the Dytiscidae family using molecular and/or morphological data (Shull et al. 2001; Miller et al. 2006; Alarie and Michat 2007; Ribera et al. 2008; Miller et al. 2009). Notably, these studies found polyphyletic groups in certain taxa of aquatic insects, particularly in Hydroporini and Dytiscinae, which is consistent with the findings of our study.

Our data suggests that Dytiscinae can be divided into three clades (Figure 3): Cybistrini, Dytiscini, and Aciliini + Eretini; the phylogenetic relationships among these three clades were consistent with those reported in a previous study on larval chaetotaxy (Alarie et al. 2011).

By addressing the mismatch between morphological traits and molecular data, our findings contribute to a better understanding of the phylogenetic relationship of the Dytiscidae family. Specifically, the analysis of the *C. brevis* mitogenome revealed that the Cybistrini tribe diverged relatively early compared to other Hydroporinae tribes, confirming previous studies and improving our understanding of the classification of Dytiscidae.

To further elucidate the phylogenetic relationships, future research should integrate molecular and morphological data and cover wider range of Dytiscidae species. Research on the taxonomy of Dytiscidae can also benefit from the integration of different phylogenetic analysis methods and data.

## Supplementary Material

Supplemental Material

Supplemental Material

## Data Availability

The genome sequence data that support the findings of this study are openly available in GenBank of NCBI at [https://www.ncbi.nlm.nih.gov] (https://www.ncbi.nlm.nih.gov/nuccore/MZ504983.1) under the accession no. MZ504983. The genome sequence data deposited in Harvard Dataverse (https://doi.org/10.7910/DVN/CCPTF6)

## References

[CIT0001] Alarie Y, Michat MC. 2007. Phylogenetic analysis of Hydroporinae (Coleoptera: dytiscidae) based on larval morphology, with description of first instar of *Laccornellus lugubris*. Ann Entomol Soc Am. 100(5):655–665. doi:10.1603/0013-8746(2007)100[655:PAOHCD]2.0.CO;2.

[CIT0002] Alarie Y, Michat MC, Miller KB. 2011. Notation of primary setae and pores on larvae of Dytiscinae (Coleoptera: dytiscidae), with phylogenetic considerations. Zootaxa. 3087(1):1–55. doi:10.11646/zootaxa.3087.1.1.

[CIT0003] Bernt M, Donath A, Jühling F, Externbrink F, Florentz C, Fritzsch G, Pütz J, Middendorf M, Stadler PF. 2013. MITOS: improved de novo metazoan mitochondrial genome annotation. Mol Phylogenet Evol. 69(2):313–319. doi:10.1016/j.ympev.2012.08.023. 22982435

[CIT0004] Cameron SL. 2014. Insect mitochondrial genomics: implications for evolution and phylogeny. Annu Rev Entomol. 59(1):95–117. doi:10.1146/annurev-ento-011613-162007. 24160435

[CIT0005] Deuve T, Cruaud A, Genson G, Rasplus JY. 2012. Molecular systematics and evolutionary history of the genus *Carabus* (Col. Carabidae). Mol Phylogenet Evol. 65(1):259–275. doi:10.1016/j.ympev.2012.06.015. 22750112

[CIT0006] Hyde J, Cooper SJ, Munguia P, Humphreys WF, Austin AD. 2018. The first complete mitochondrial genomes of subterranean dytiscid diving beetles (Limbodessus and Paroster) from calcrete aquifers of Western Australia. Aust J Zool. 65(5):283–291. doi:10.1071/ZO17076.

[CIT0007] Jäch MA, Balke M. 2008. Global diversity of water beetles (Coleoptera) in freshwater. Hydrobiologia. 595(1):419–442. doi:10.1007/s10750-007-9117-y.

[CIT0008] Kim SR, Kim JS, Kim KY, Kim MJ, Jeong JS, Kim I. 2017. Complete mitochondrial genome of the wild silkmoth, *Saturnia boisduvalii* (Lepidoptera: Saturniidae). Entomol Res. 47(6):344–351. doi:10.1111/1748-5967.12256.

[CIT0009] Laslett D, Canbäck B. 2008. ARWEN: a program to detect tRNA genes in metazoan mitochondrial nucleotide sequences. Bioinformatics. 24(2):172–175. doi:10.1093/bioinformatics/btm573. 18033792

[CIT0010] Linard B, Crampton-Platt A, Moriniere J, Timmermans MJTN, Andújar C, Arribas P, Miller KE, Lipecki J, Favreau E, Hunter A, et al. 2018. The contribution of mitochondrial metagenomics to large-scale data mining and phylogenetic analysis of Coleoptera. Mol Phylogenet Evol. 128:1–11. doi:10.1016/j.ympev.2018.07.008. 30055354

[CIT0011] Liu Y, Dong Y, Ye Z, Wu S, Li G. 2023. The complete mitochondrial genome of *Anthrenus museorum* (Coleoptera: bostrichiformia: dermestidae) from China. Mitochondrial DNA B Resour. 8(3):405–409. doi:10.1080/23802359.2023.2187655. 37426905 PMC10324981

[CIT0012] Lowe TM, Eddy SR. 1997. tRNAscan-SE: a program for improved detection of transfer RNA genes in genomic sequence. Nucleic Acids Res. 25(5):955–964. doi:10.1093/nar/25.5.955. 9023104 PMC146525

[CIT0013] Michat MC. 2010. Descriptions of larvae of Megadytes (Coleoptera: dytiscidae: dytiscinae): the subgenera *Trifurcitus* and *Megadytes* s. str., ground plan of chaetotaxy of the genus and phylogenetic analysis. Eur J Entomol. 107(3):377–392. 2010. doi:10.14411/eje.2010.047.

[CIT0014] Michat MC, Alarie Y, Miller KB. 2017. Higher‐level phylogeny of diving beetles (Coleoptera: dytiscidae) based on larval characters. Syst Entomol. 42(4):734–767. doi:10.1111/syen.12243.

[CIT0015] Michat MC, Torres PL. 2009. A preliminary study on the phylogenetic relationships of *Copelatus* Erichson (Coleoptera: dytiscidae: copelatinae) based on larval chaetotaxy and morphology. Hydrobiologia. 632(1):309–327. doi:10.1007/s10750-009-9853-2.

[CIT0016] Miller KB, Bergsten J, Whiting MF. 2009. Phylogeny and classification of the tribe Hydaticini (Coleoptera: dytiscidae): partition choice for Bayesian analysis with multiple nuclear and mitochondrial protein‐coding genes. Zool Scr. 38(6):591–615. doi:10.1111/j.1463-6409.2009.00393.x.

[CIT0017] Miller KB, Wolfe GW, Biström O. 2006. Phylogeny of the Hydroporinae and classification of the genus *Peschetius* Guignot, 1942 (Coleoptera: dytiscidae). Insect Syst Evol. 37:257–279.

[CIT0018] Nilsson AN, Hájek J. 2023. A World Catalogue of the Family Dytiscidae or the Diving Beetles (Coleoptera, Adephaga). 2023 pp:79–85. Version 1.I.

[CIT0019] Ribera I, Vogler AP, Balke M. 2008. Phylogeny and diversification of diving beetles (Coleoptera: dytiscidae). Cladistics. 24(4):563–590. doi:10.1111/j.1096-0031.2007.00192.x. 34879635

[CIT0020] Ronquist F, Teslenko M, Van Der Mark P, Ayres DL, Darling A, Höhna S, Larget B, Liu L, Suchard MA, Huelsenbeck JP. 2012. MrBayes 3.2: efficient Bayesian phylogenetic inference and model choice across a large model space. Syst Biol. 61(3):539–542. doi:10.1093/sysbio/sys029. 22357727 PMC3329765

[CIT0021] Shull VL, Vogler AP, Baker MD, Maddison DR, Hammond PM. 2001. Sequence alignment of 18S ribosomal RNA and the basal relationships of adephagan beetles: evidence for monophyly of aquatic families and the placement of Trachypachidae. Syst Biol. 50(6):945–969. doi:10.1080/106351501753462894. 12116642

[CIT0022] Thompson JD, Gibson TJ, Plewniak F, Jeanmougin F, Higgins DG. 1997. The Clustal-X Windows interface: flexible strategies for multiple sequence alignment aided by quality analysis tools. Nucleic Acids Res. 25(24):4876–4882. doi:10.1093/nar/25.24.4876. 9396791 PMC147148

[CIT0023] Timmermans MJ, Barton C, Haran J, Ahrens D, Culverwell CL, Ollikainen A, Dodsworth S, Foster PG, Bocak L, Vogler AP. 2016. Family-level sampling of mitochondrial genomes in Coleoptera: compositional heterogeneity and phylogenetics. Genome Biol Evol. 8(1):161–175. doi:10.1093/gbe/evv241. PMC4758238 26645679

[CIT0024] Vasconcelos S, Oliveira RR, Pires ES, Pietrobon T, Prous X, Asenjo A, Oliveira G. 2021. Complete mitochondrial genome of a cave dwelling *Desmopachria* (Insecta: coleoptera: dytiscidae) from the Eastern Amazon. Mitochondrial DNA B Resour. 6(2):415–417. doi:10.1080/23802359.2020.1870885. 33659697 PMC7872522

