# Supplementary figures and images for: Complete mitochondrial genome of the diving beetle, Cybister brevis Aubé, 1838 (Coleoptera, Dytiscidae) from Jeju Island
Source: Mitochondrial DNA B Resour. 2024 Feb 22;9(2):295–9. doi: 10.1080/23802359.2024.2317327 (PMC10885740; doi:10.1080/23802359.2024.2317327)

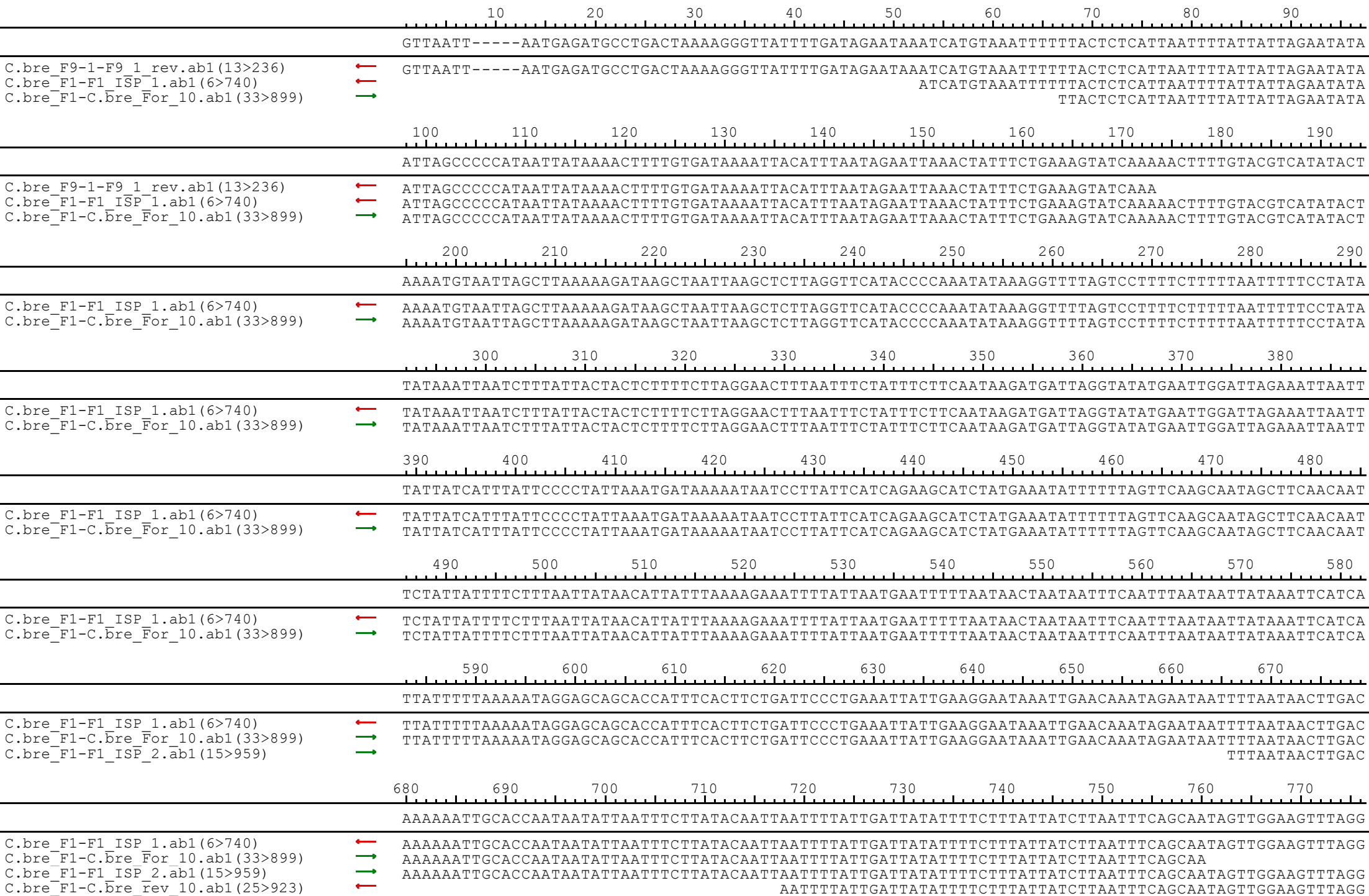

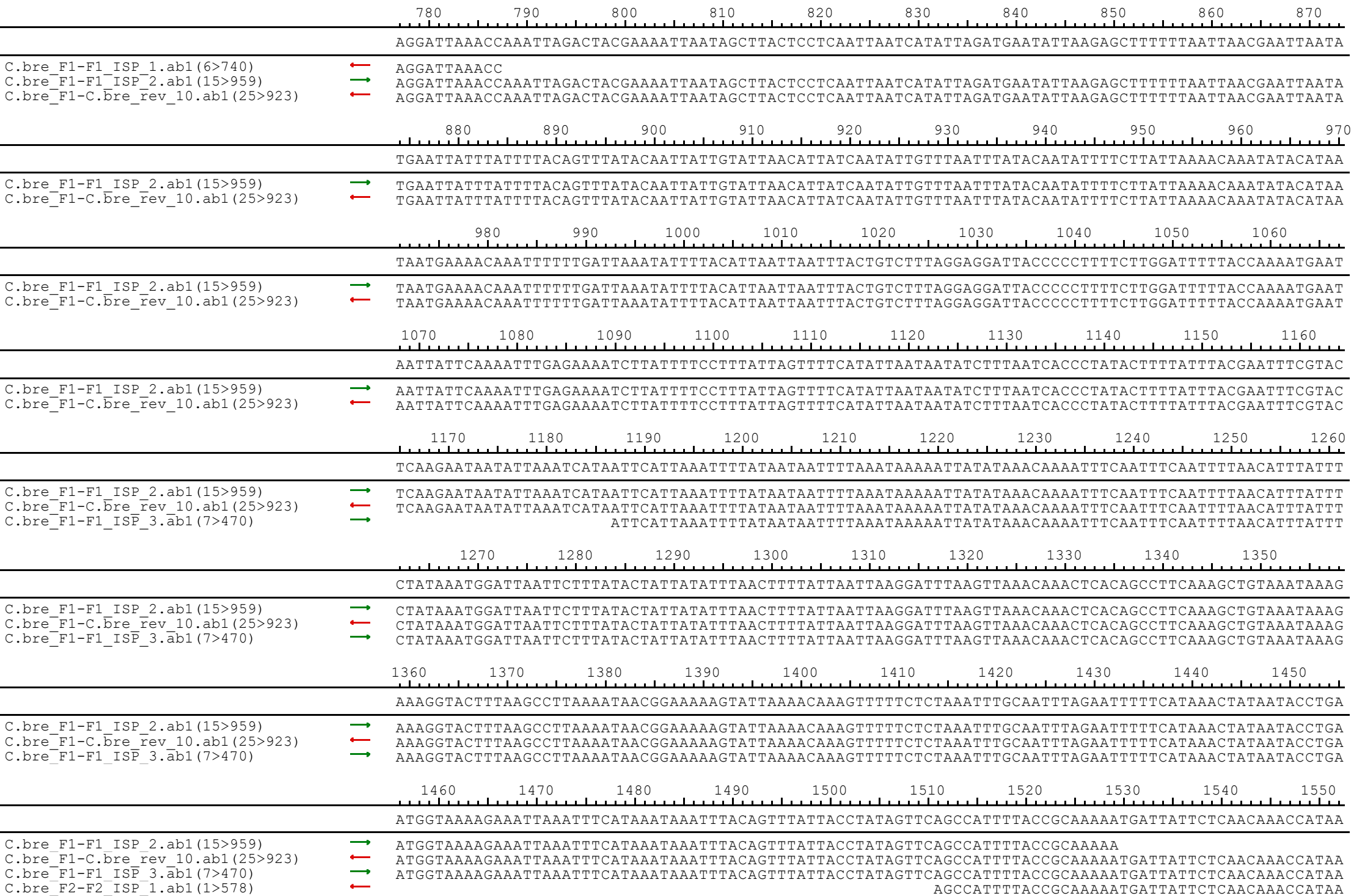

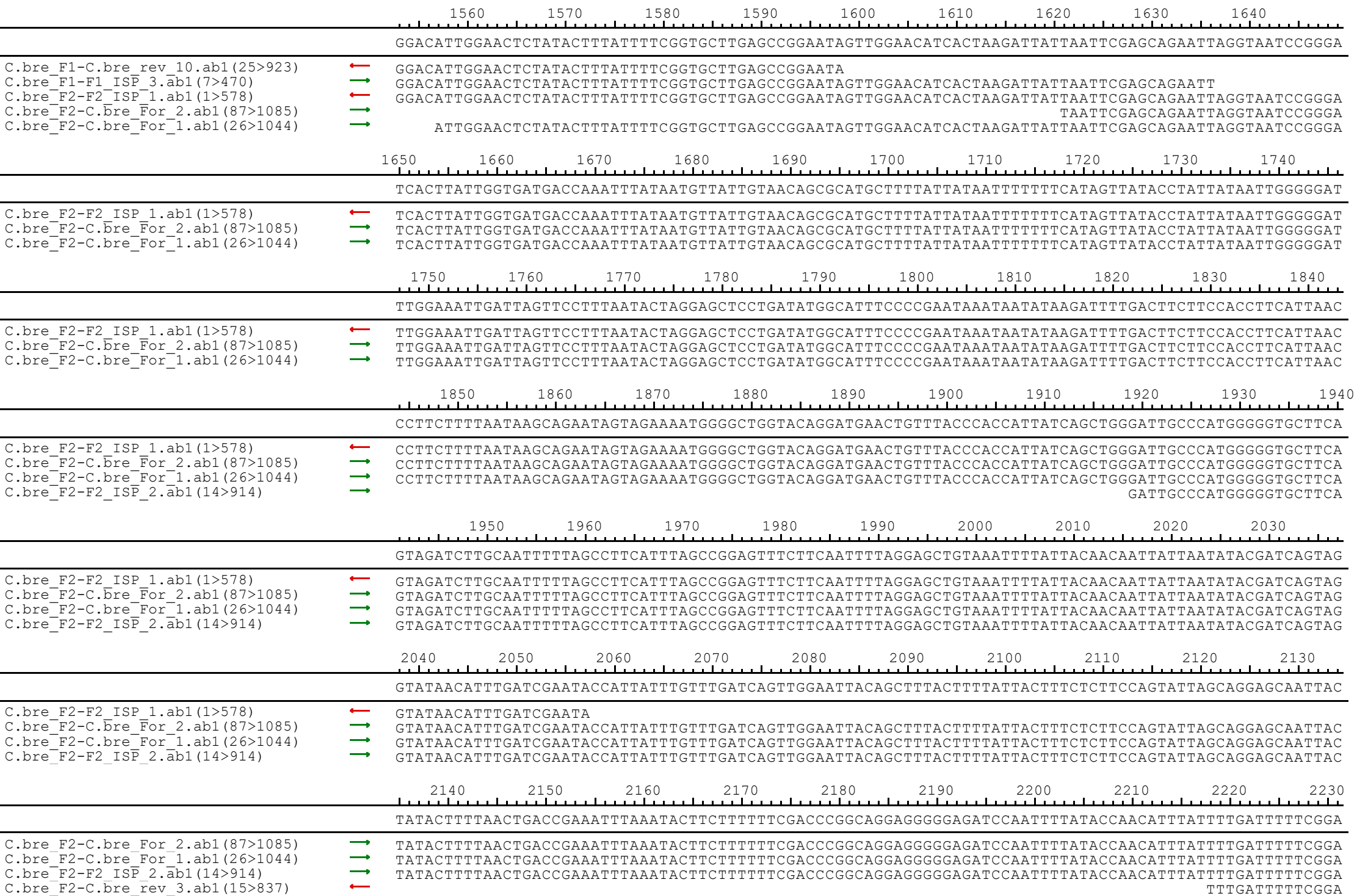

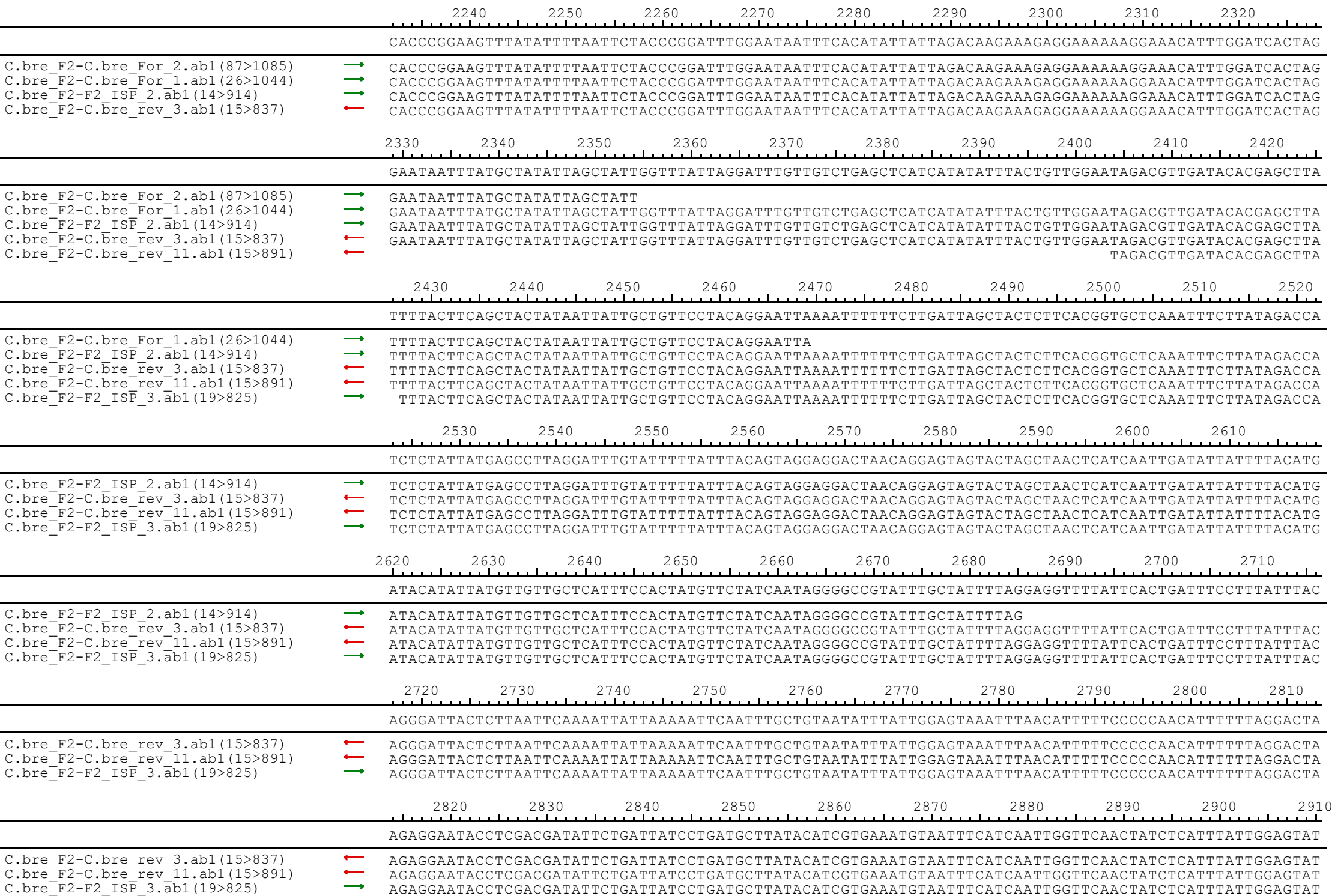

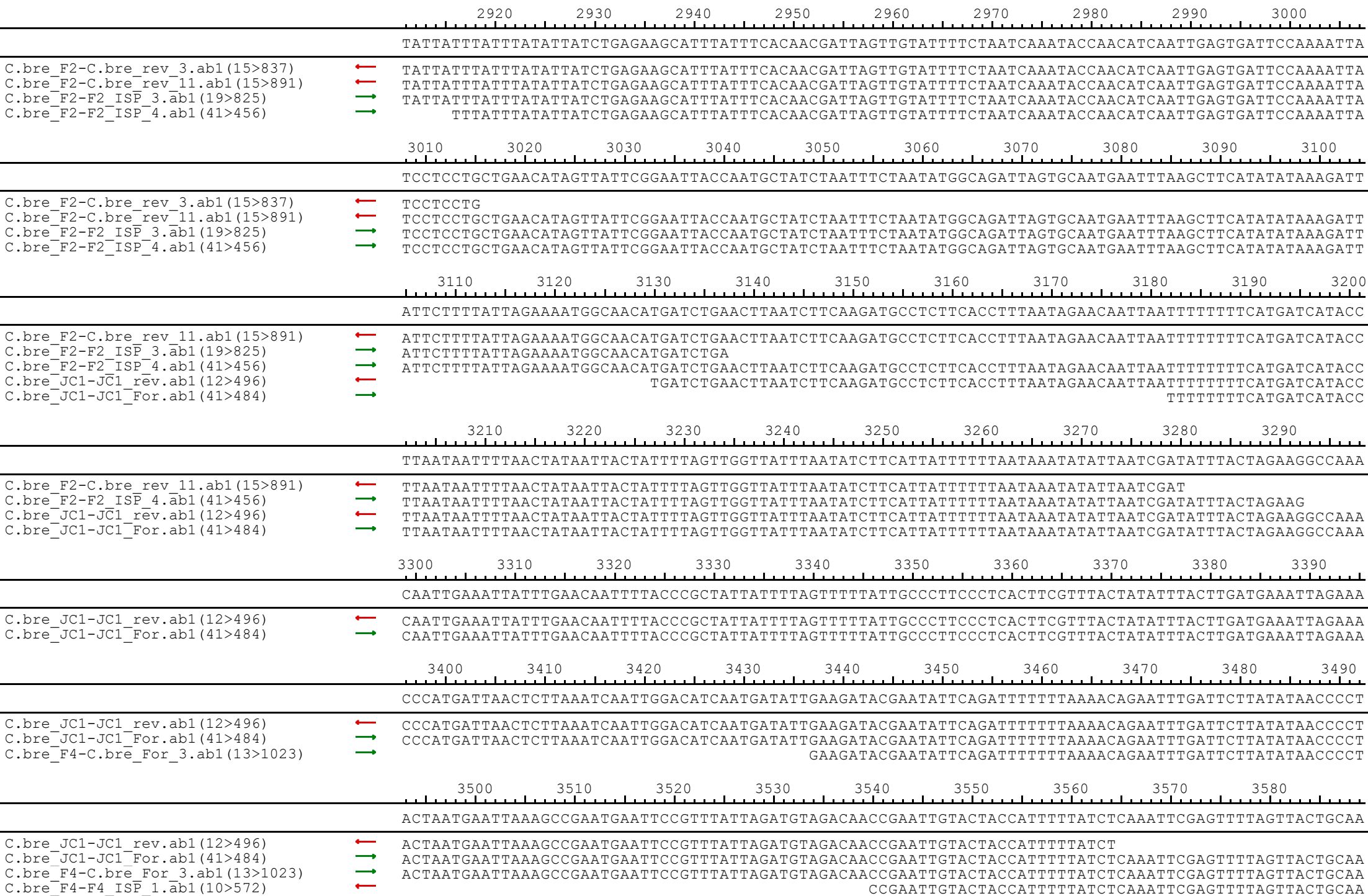

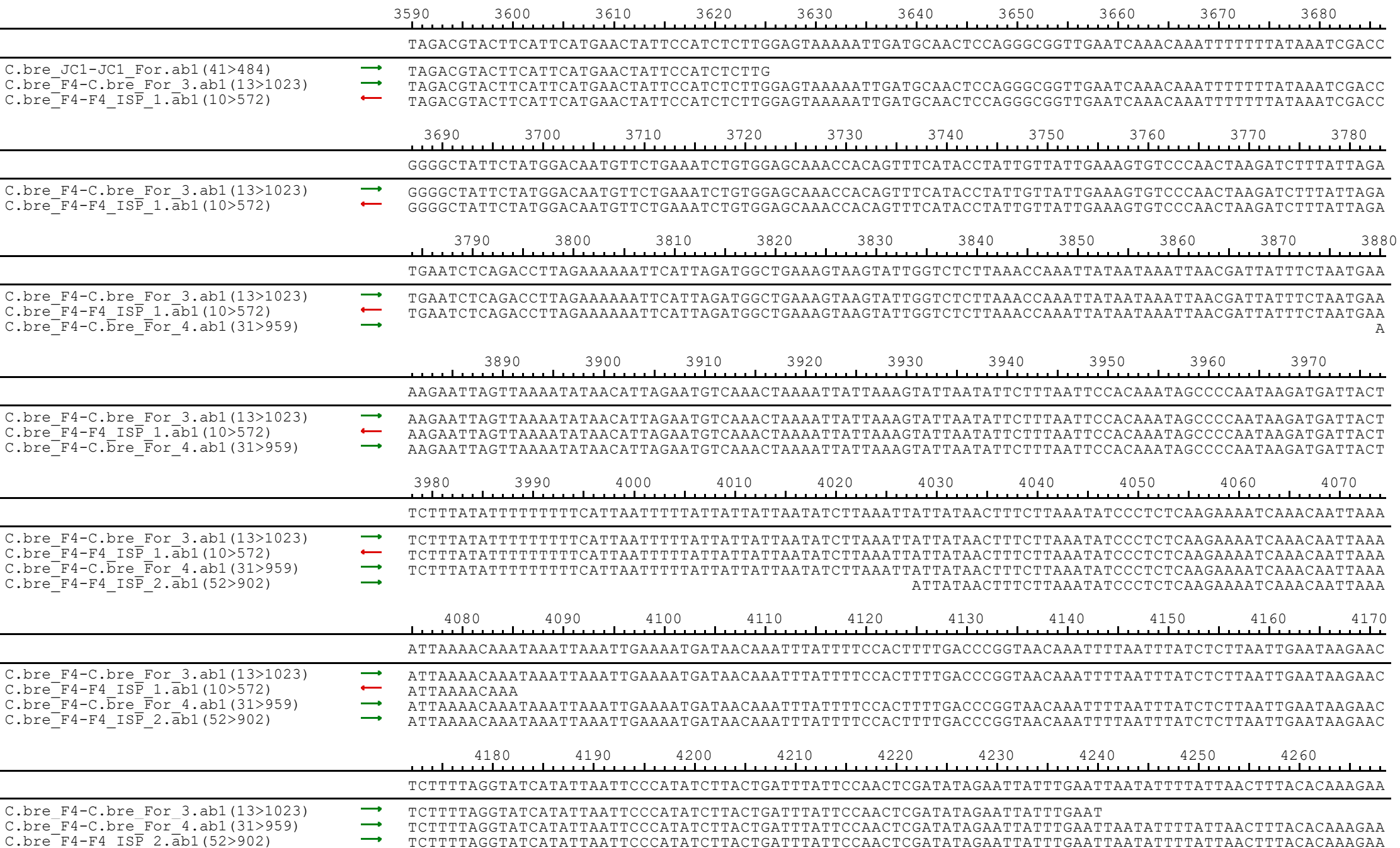

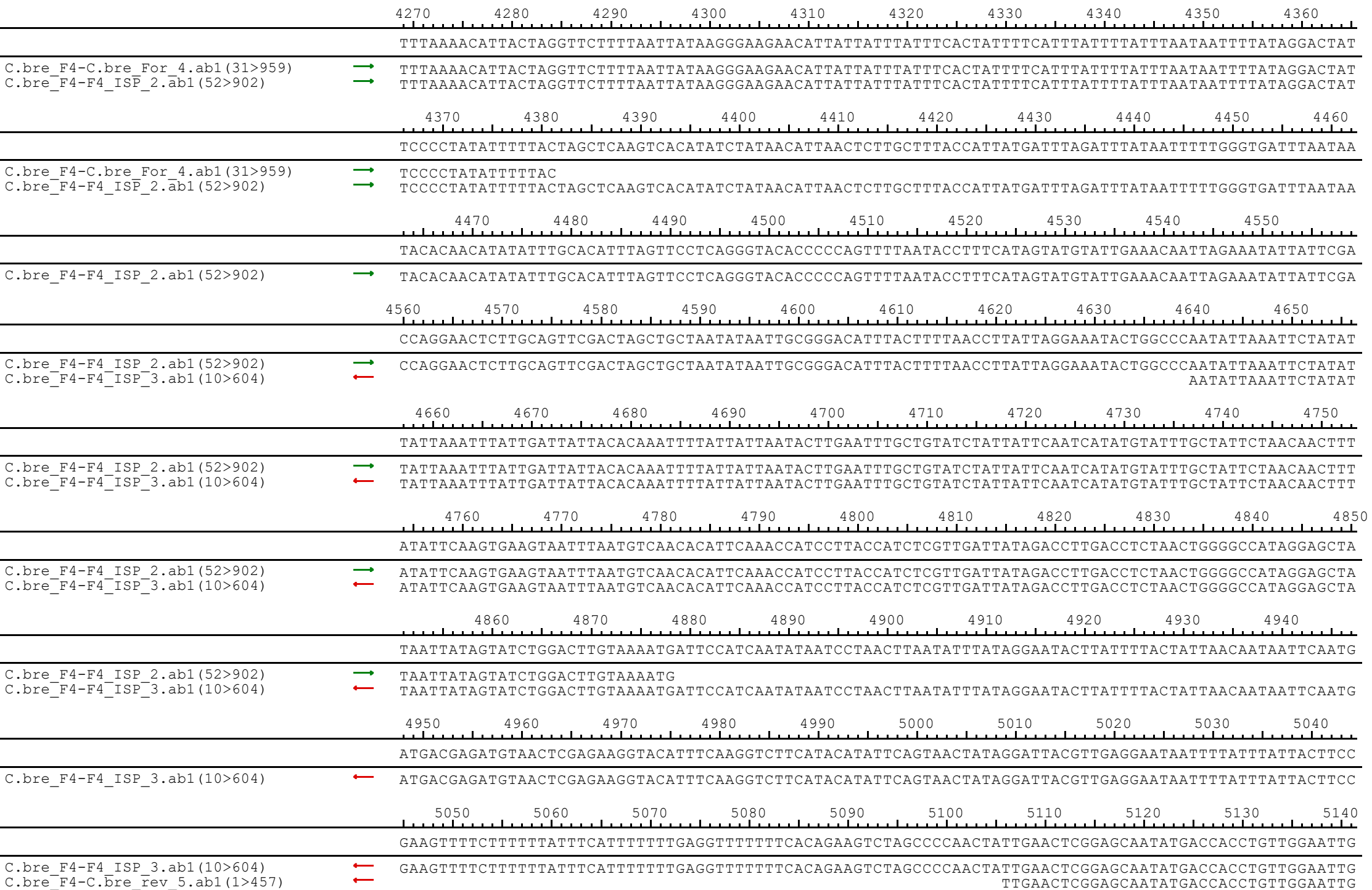

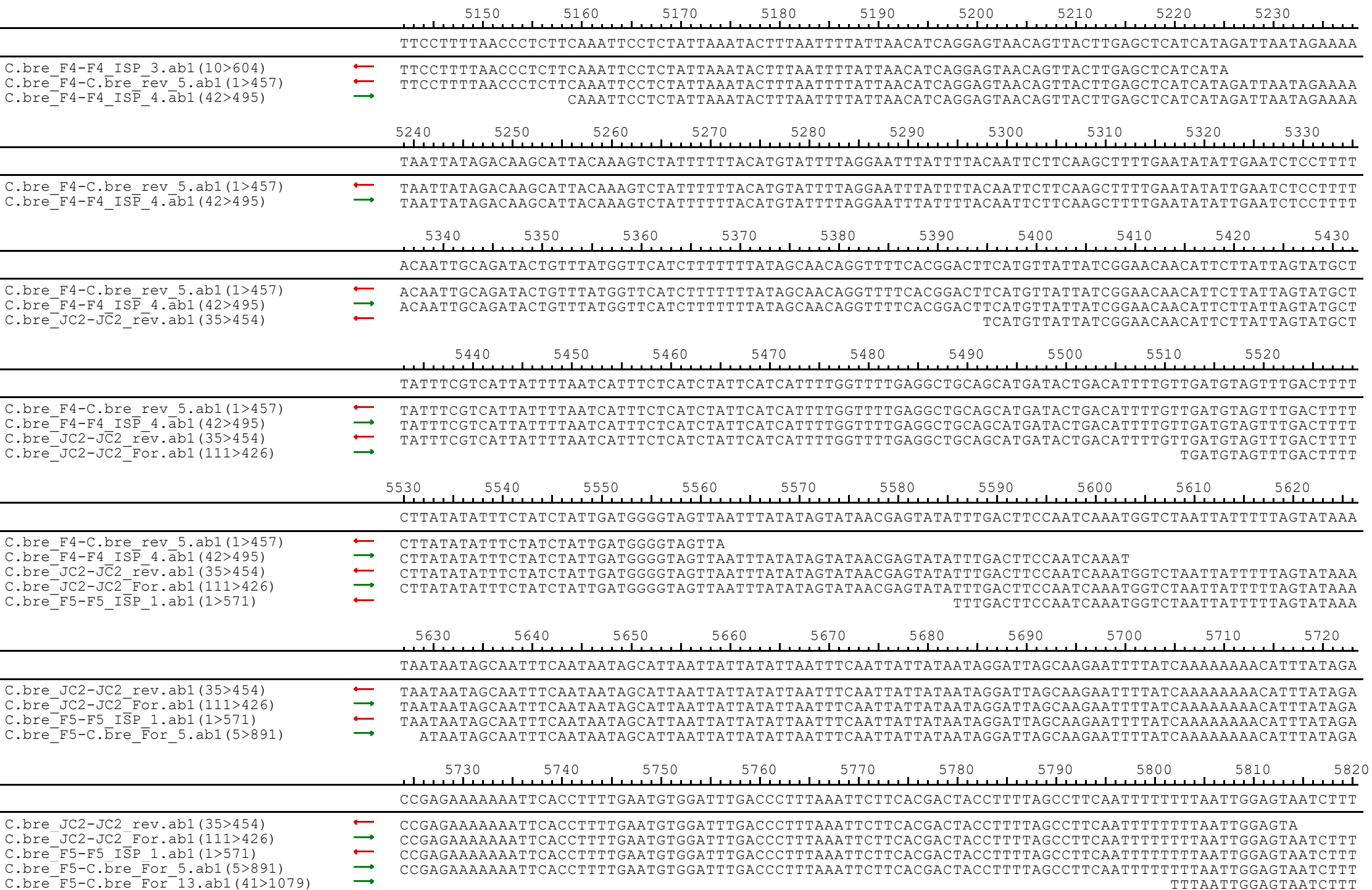

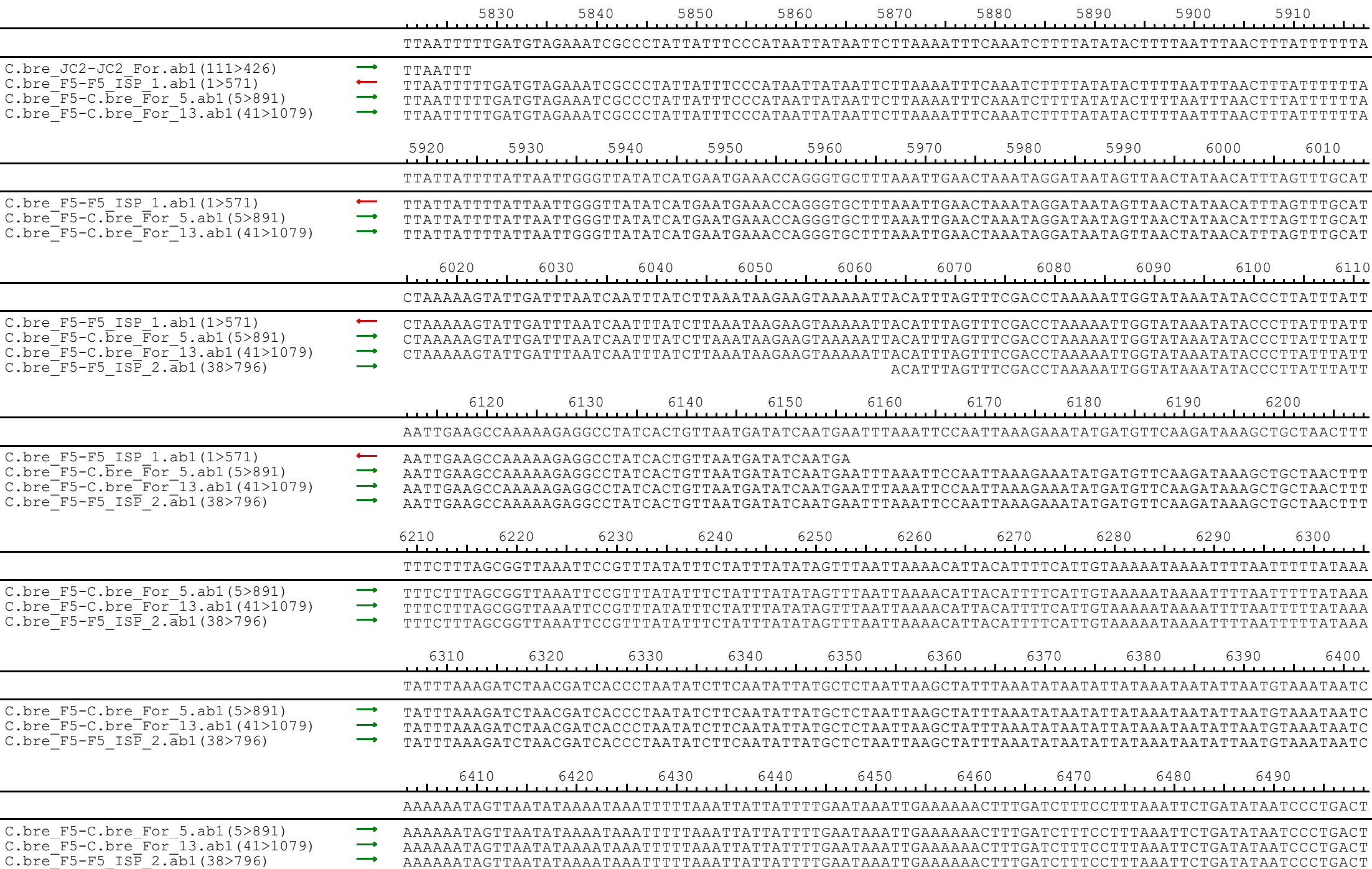

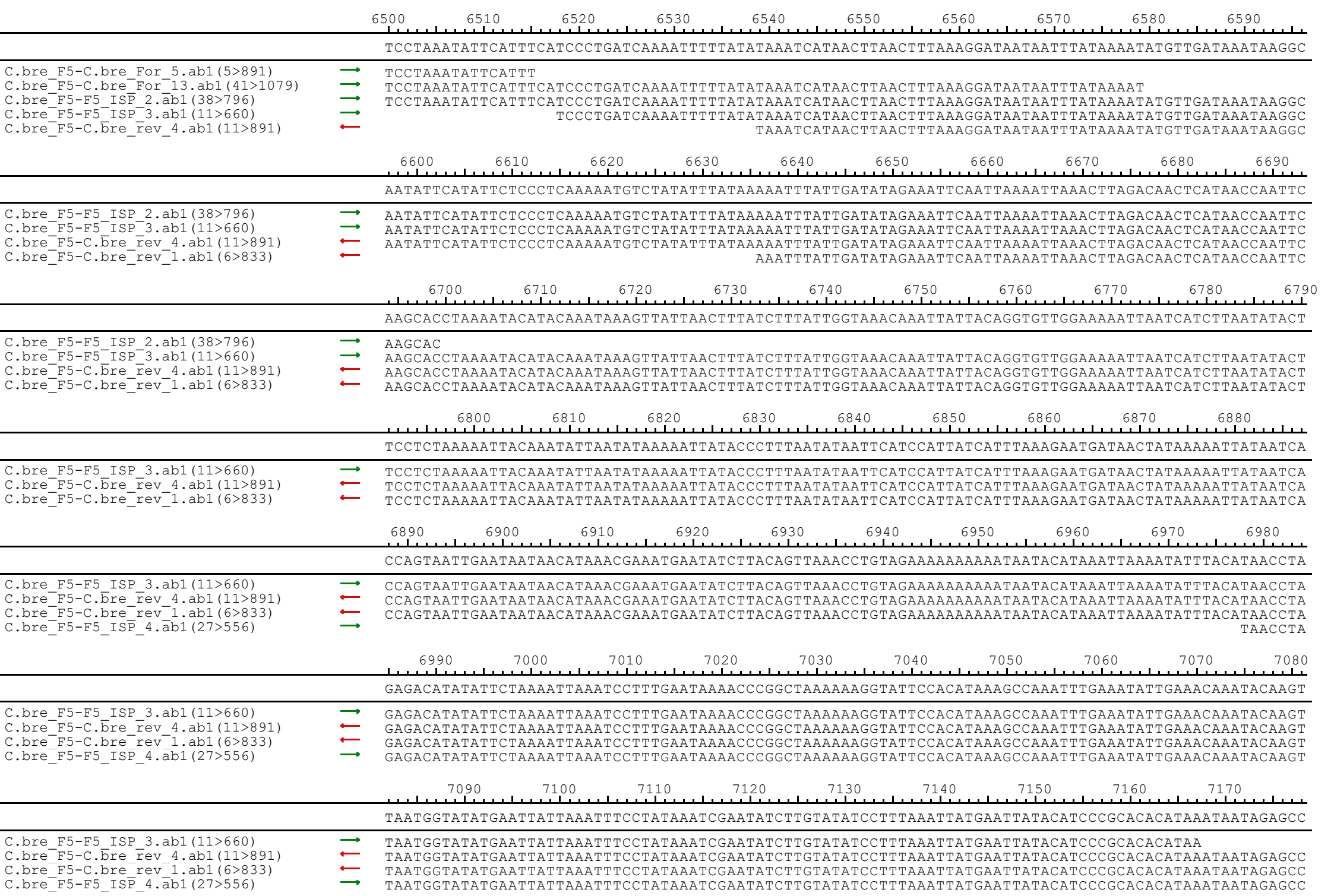

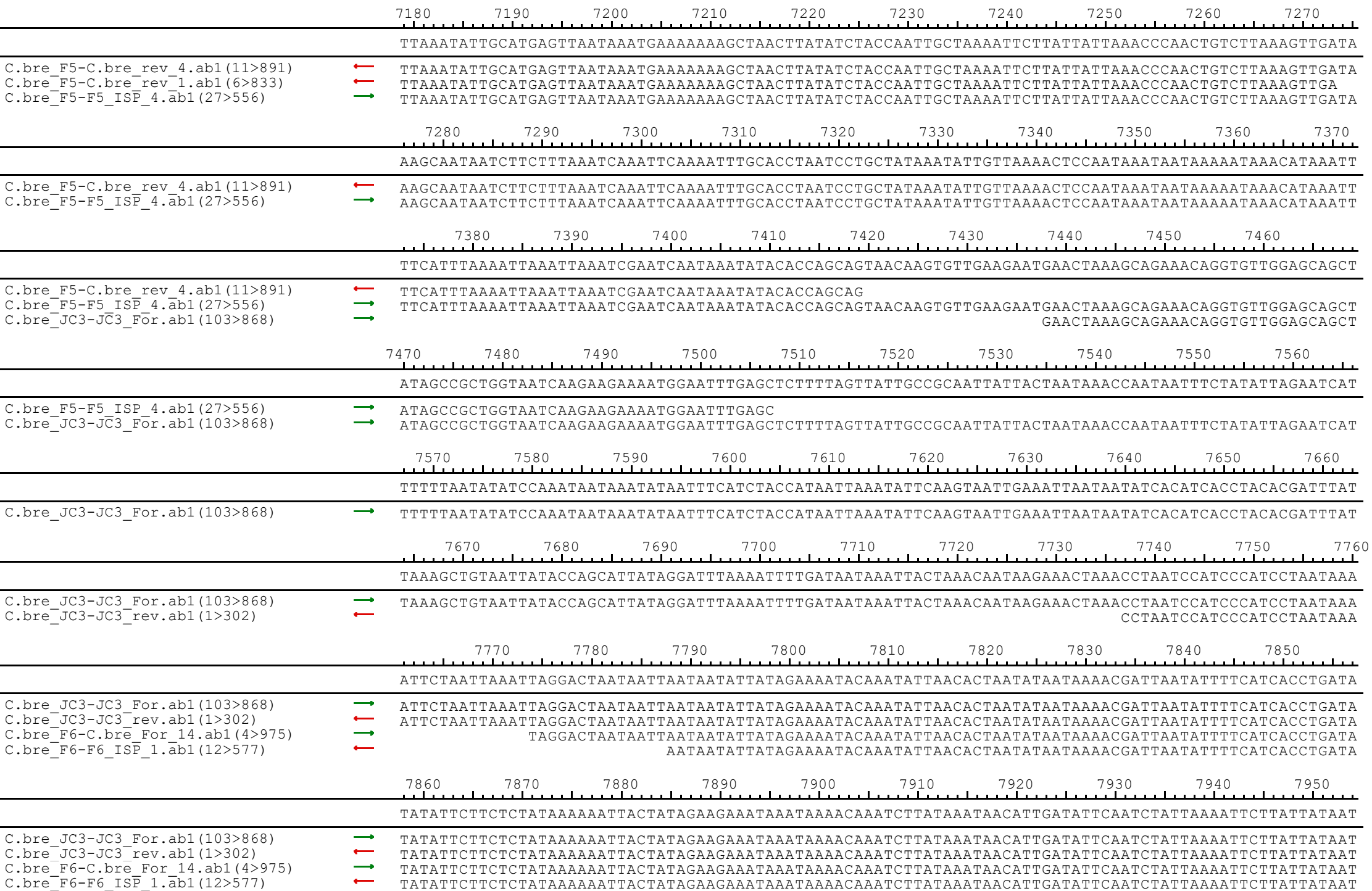

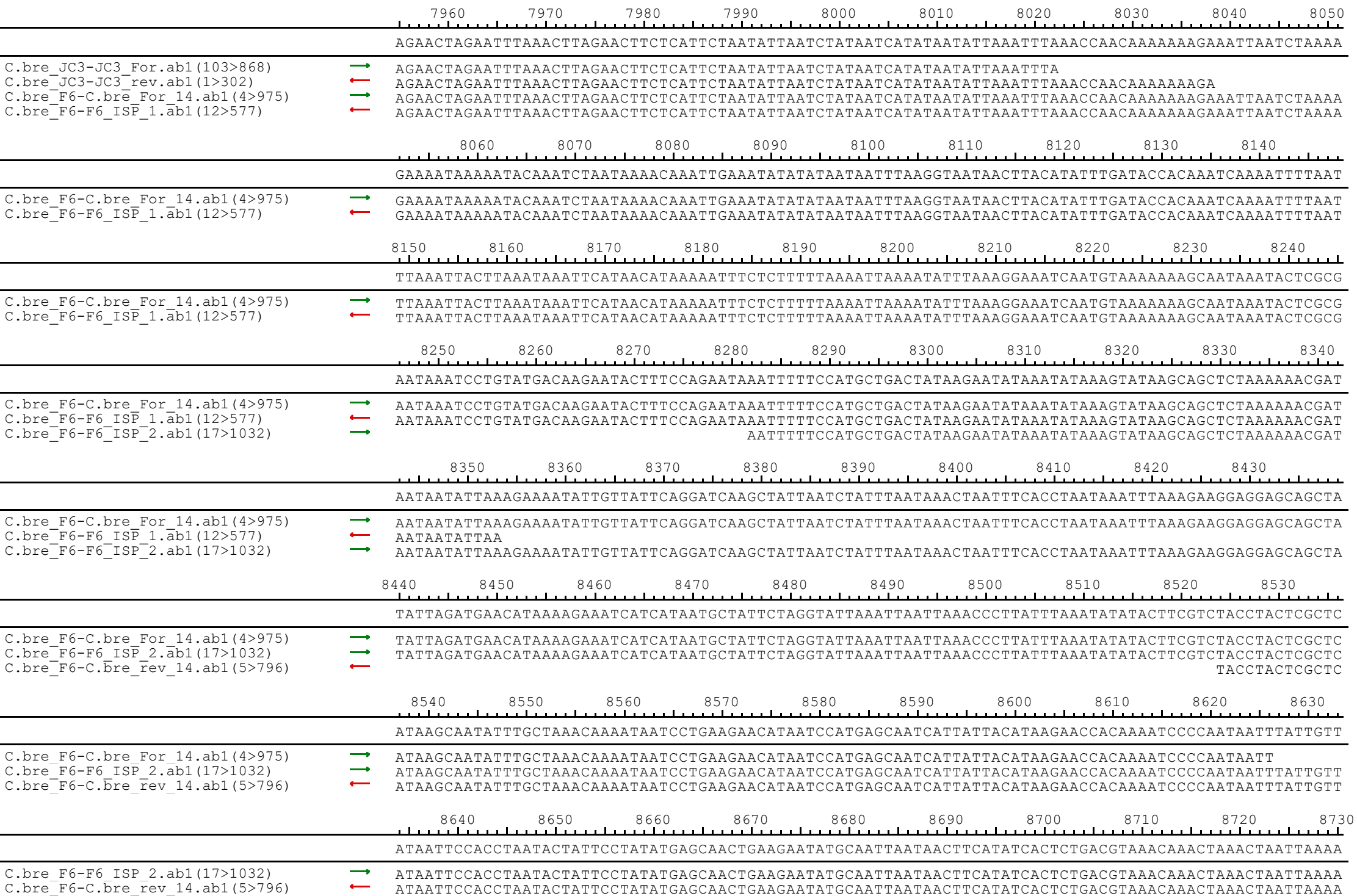

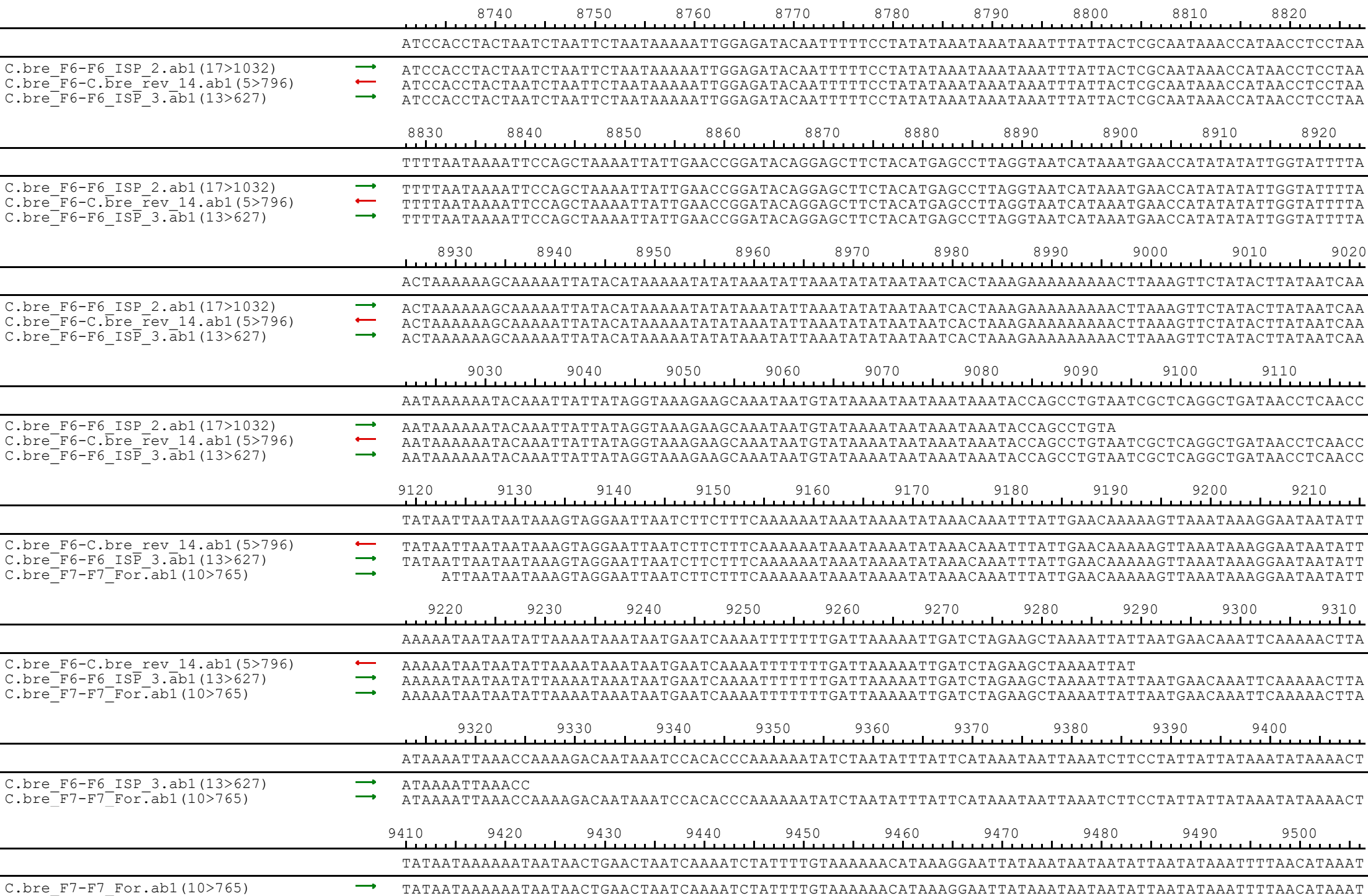

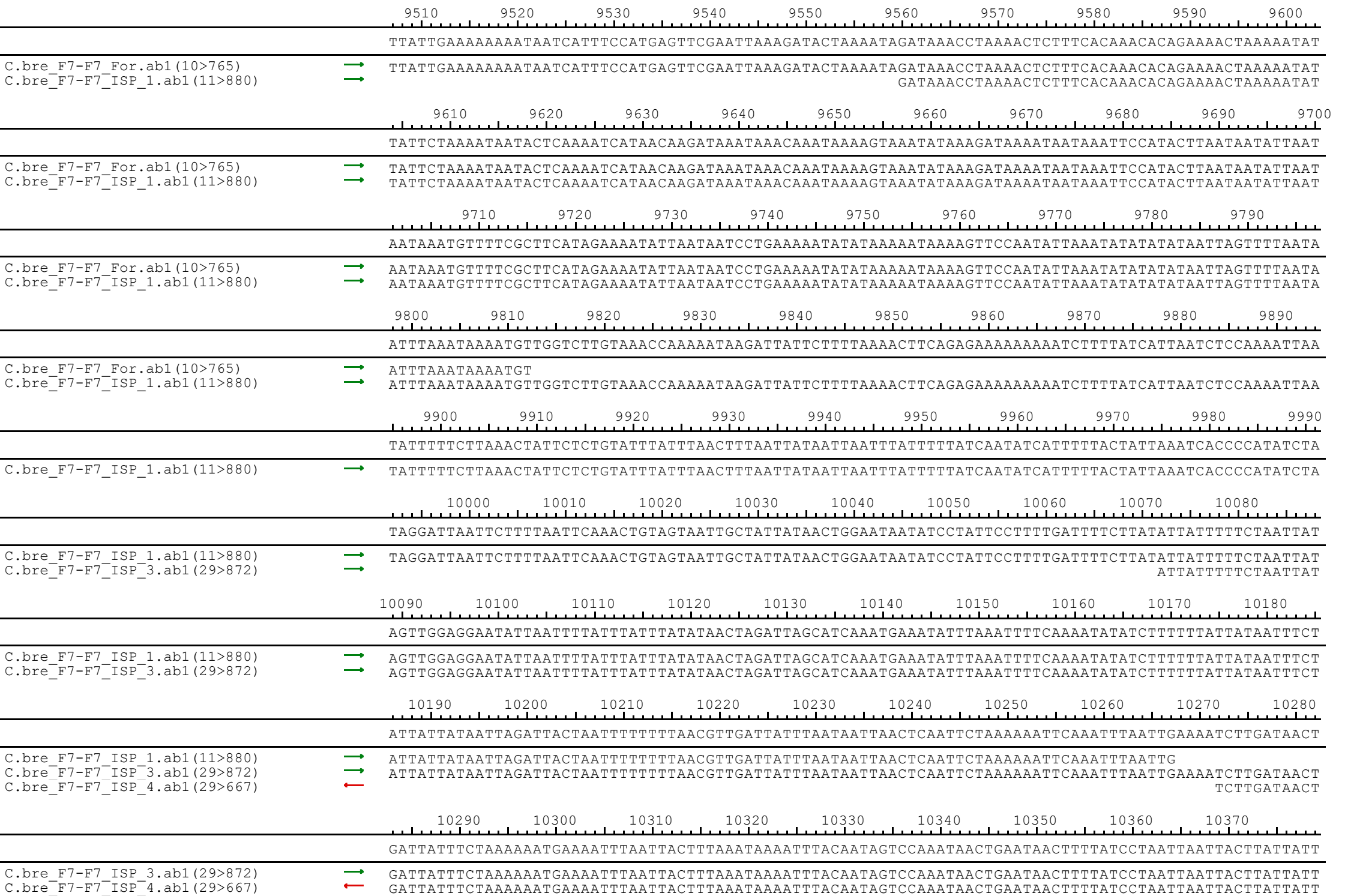

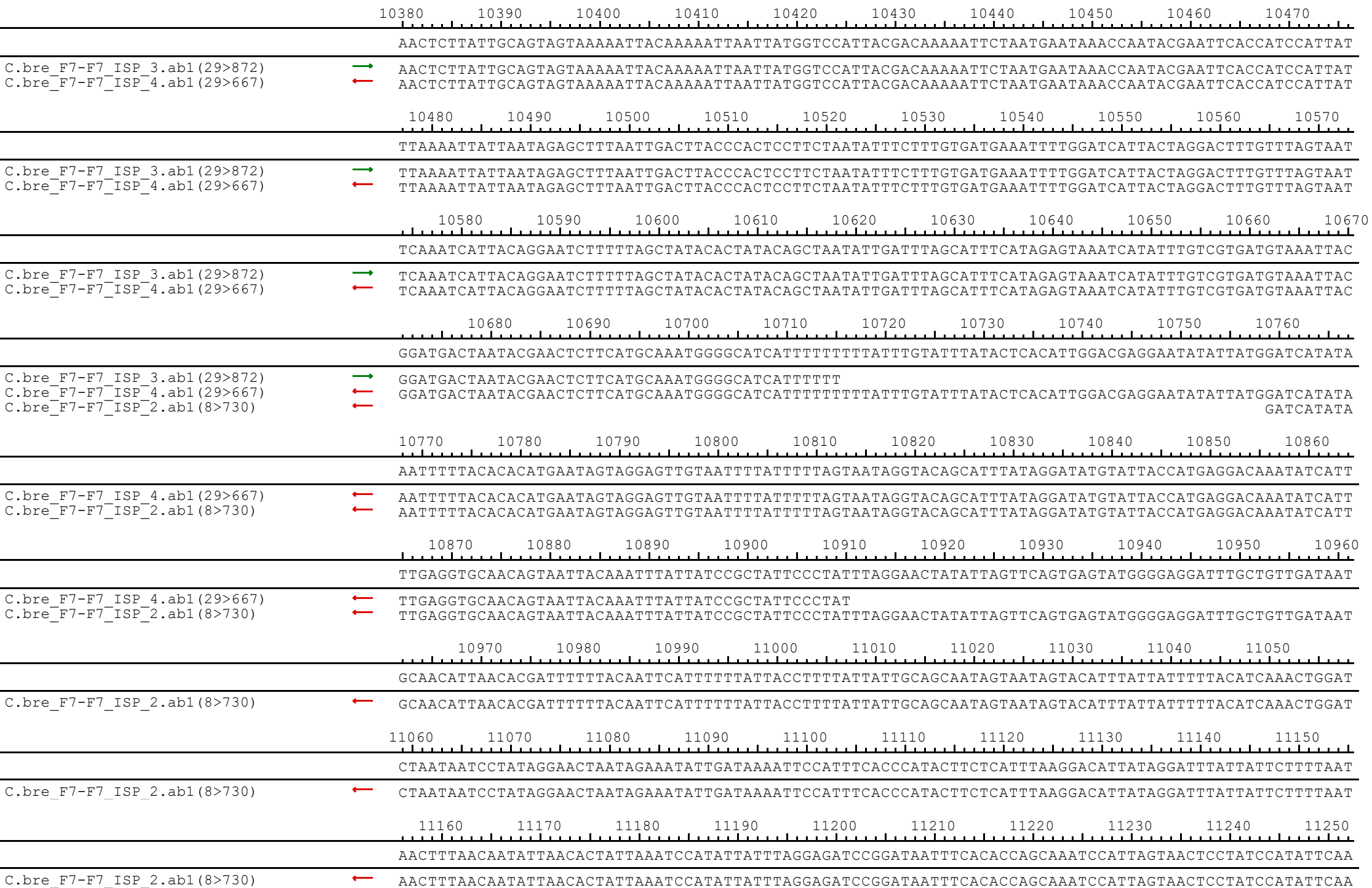

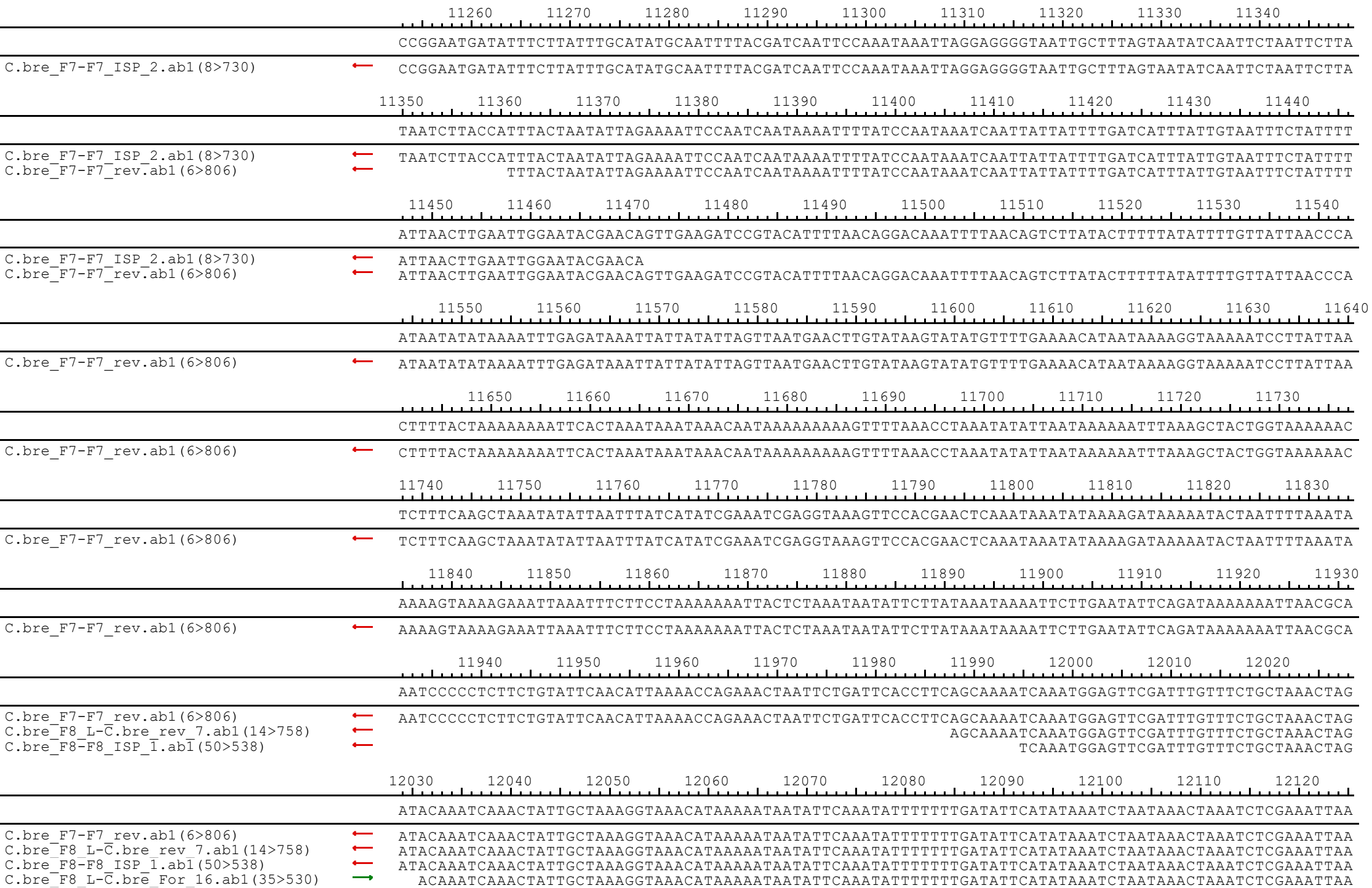



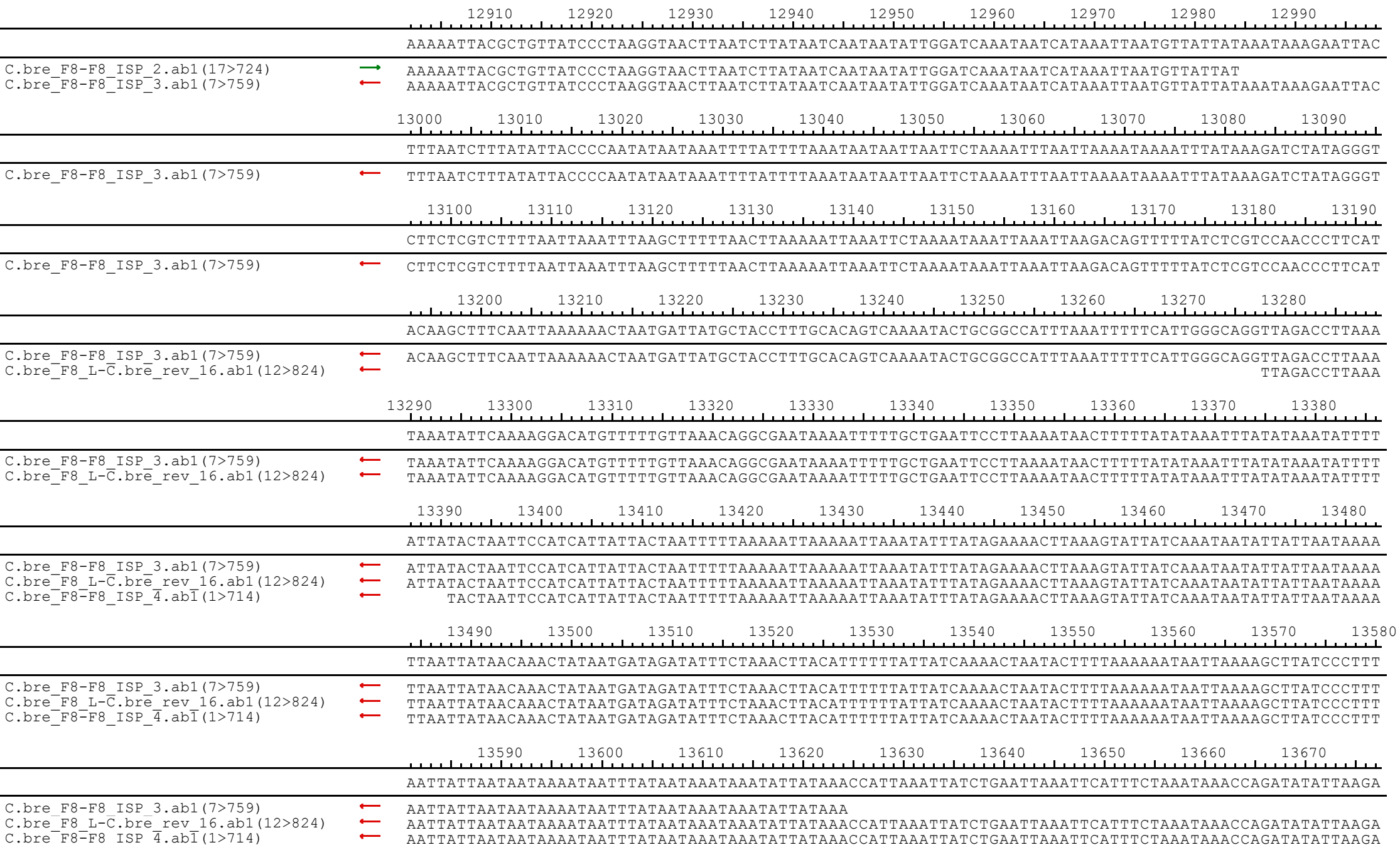

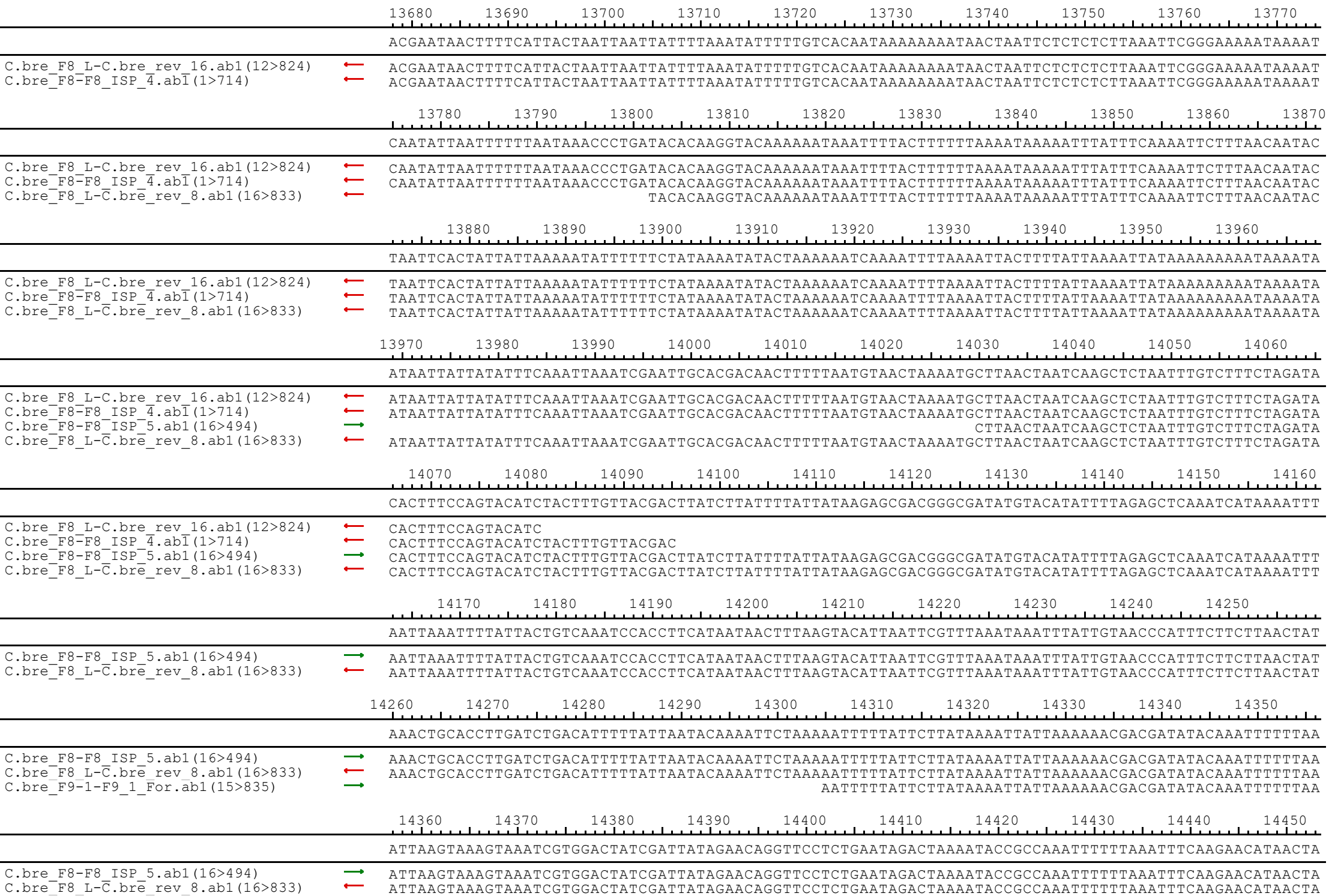

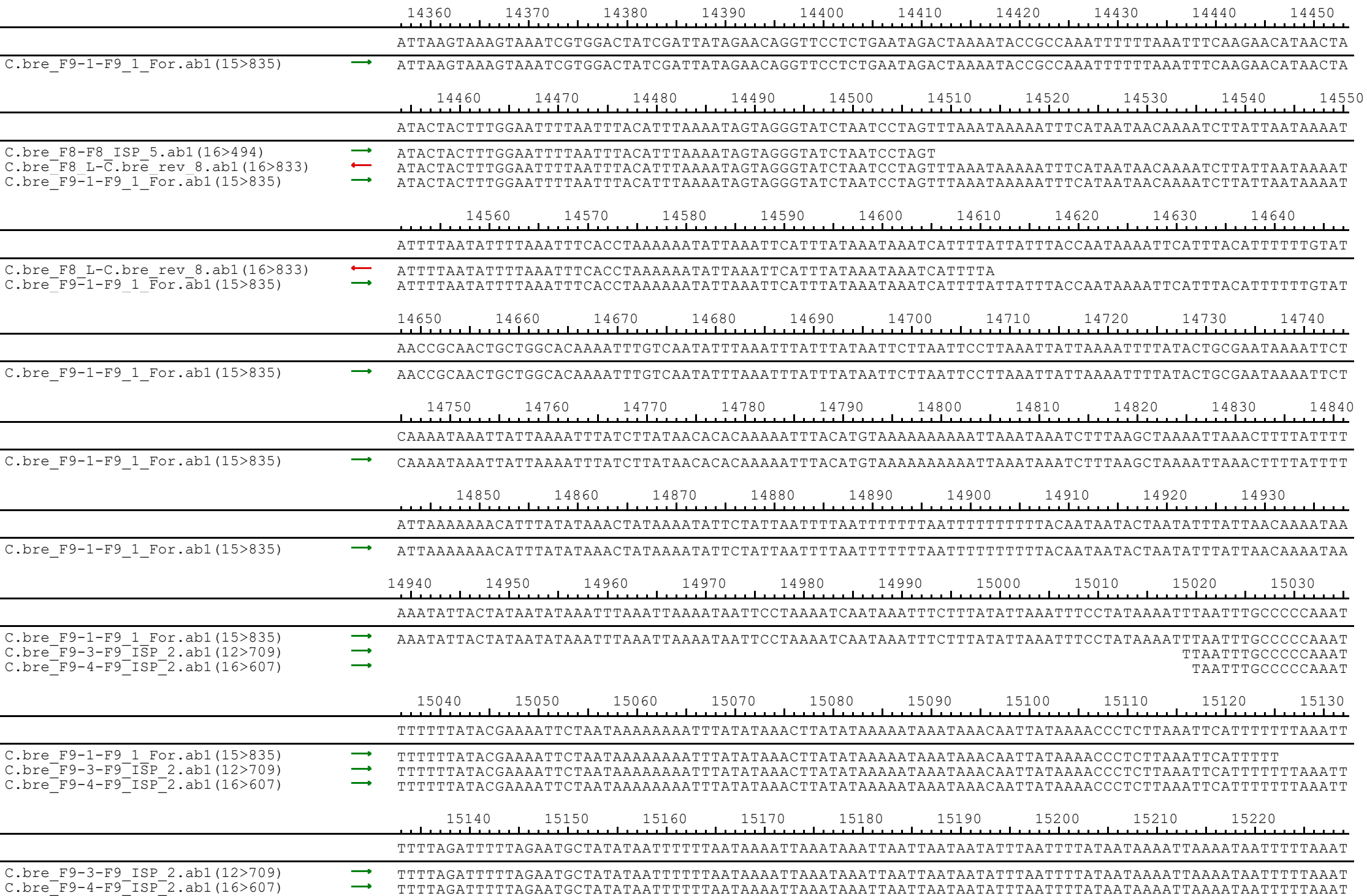

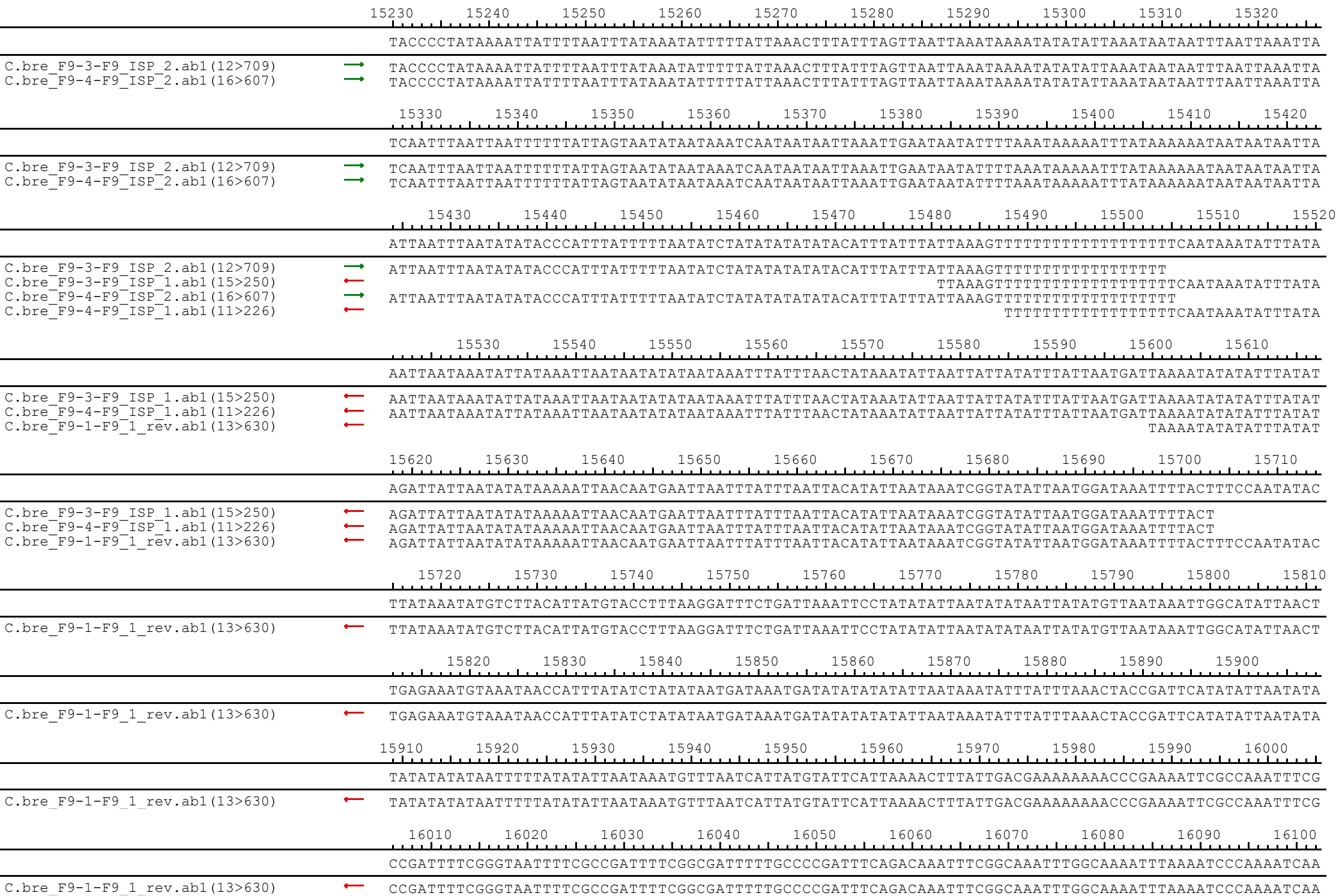

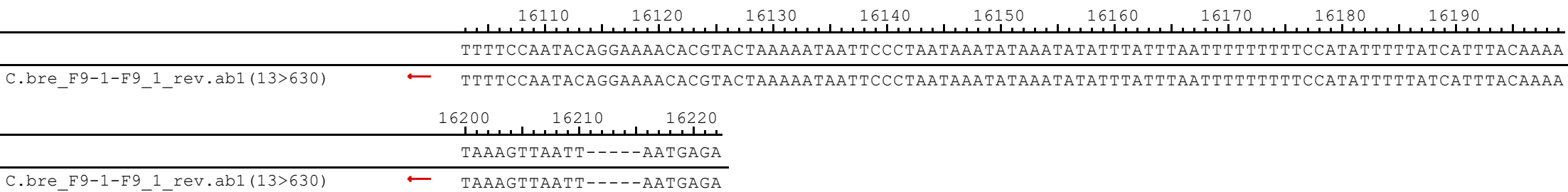

Supplement: Supplemental Material [file TMDN_A_2317327_SM4410.pdf]
