# Supplementary material for: Complete mitochondrial genome of the diving beetle, Cybister brevis Aubé, 1838 (Coleoptera, Dytiscidae) from Jeju Island
Source: Mitochondrial DNA B Resour. 2024 Feb 22;9(2):295–9. doi: 10.1080/23802359.2024.2317327 (PMC10885740; doi:10.1080/23802359.2024.2317327)
Supplement: Supplemental Material [file TMDN_A_2317327_SM4259.pdf]

Mitochondrial DNA Full Sequencing : *Cybister brevis*

Primer name	Sequence	PCR	Seq.
C.bre_For_10	GATTAAAATGAGATGCCTGAAAAA	O	O
C.bre_rev_10	ACCCGGGTTTCCTAATTCTG	O	O
F1_ISP_1	TGATTAATTGAGGAGTAAGC		O
F1_ISP_2	ATTCCCTGAAATTATTGAAG		O
F1_ISP_3	TTACGAATTTCGTACTCAAG		O
C.bre_For_1	CCTATAATTCAGCCATTTTACCG	O	O
C.bre_For_2	TGATTATTTTCAACAAACCATAAGGA	O	O
C.bre_rev_11	TGCGGGTAGAATTGTTCAAA	O	O
C.bre_rev_3	TTCATTGCACTAATCTGCCATA	O	O
F2_ISP_1	GCTAATACTGGAAGAGAAAG		O
F2_ISP_2	CTGGTACAGGATGAACTG		O
F2_ISP_3	TCATCATATATTTACTGTTG		O
F2_ISP_4	TGCTTATACATCGTGAAATG		O
C.bre_For_3	CTCCAGGCCGATTAAATCAA	O	O
C.bre_For_4	TGAAAGTAAGTATTGGTCTCTTAAACC	O	O
C.bre_rev_5	AATTAGACCATTTGATTGGAAGTC	O	O
F4_ISP_1	TGTTACCGGGTCAAAAGTGG		O
F4_ISP_2	CCCAATAAGATGATTACTTC		O
F4_ISP_3	TAGACTTTGTAATGCTTGTC		O
F4_ISP_4	AAGTCTAGCCCCAACTATTG		O
C.bre_For_13	CCCCATTTGAATGTGGATTT	O	O
C.bre_For_5	TTTGACTTCCAATCAAATGGTC	O	O
C.bre_rev_1	GAGCTCAAATTCCATTTTCTTC	O	O
C.bre_rev_4	CCAACCCCTGTTTCTGCTT	O	O
F5_ISP_1	TTAGCAGCTTTATCTTGAAC		O
F5_ISP_2	CTATAACATTTAGTTTGCATC		O
F5_ISP_3	GATCTTTCCTTTAAATTCTG		O
F5_ISP_4	TCTTACAGTTAAACCTGTAG		O
C.bre_For_14	ACCCTAATCCATCCCAACCT	O	O
C.bre_rev_14	GGGGGTGATTTATTATCATTTGG	O	O
F6_ISP_1	ATAGATTAATAGCTTGATCC		O
F6_ISP_2	AAATACTCGCGAATAAATCC		O
F6_ISP_3	TAACTTCATATCACTCTGAC		O

Primer name	Sequence	PCR	Seq.
Cbre_F7_For	ACCAGCCTGTAATCGCTCAG	O	O
Cbre_F7_rev	CGTGCTGTTGCTCAGTCAAT	O	O
F7_ISP_1	TAATCATTTCCATGAGTTCG		O
F7_ISP_2	GACTGTTAAAATTTGTCCTG		O
F7_ISP_3	GTAATTGCTATTATAACTGG		O
F7_ISP_4	AATGTTGCATTATCAACAGC		O
C.bre_For_16	CTGATTCCCCTTCAGCAAAA	O	O
C.bre_rev_16	ACATATCGCCCGTCACTCTT	O	O
C.bre_rev_7	TGTAAGCCAGGTCGGTTTCT	O	O
C.bre_rev_8	CCAGCAGTTGCGGTTATACA	O	O
F8_ISP_1	TGTGTTTTAGTTGGGGTAGC		O
F8_ISP_2	AGGAAATGTTTGTTCCTTAG		O
F8_ISP_3	ATCTGGTTTATTTAGAAATG		O
F8_ISP_4	TTGAGCTCTAAAATATGTAC		O
F8_ISP_5	ATTAAATCGAATTGCACGAC		O
Cbre_F9_I_For	AAACTGCACCTTGATCTGACA	O	O
Cbre_F9_I_rev	GGACTAAAACCTTTATATTTGGGGTA	O	O
Cbre_F9_O_For	TATAAGAGCGACGGGCGATA	O	O
Cbre_F9_O_rev	AGATGCTTCTGATGAATAAGGATT	O	O
F9_ISP_1	TCAGAAATCCTTAAAGGTAC		O
F9_ISP_2	TTCCTAAAATCAATAAATTTC		O
Cbre_JC1_For	GAAAATGGCAACATGATCTGAA	O	O
Cbre_JC1_rev	CCCTGGAGTTGCATCAATTT	O	O
Cbre_JC2_For	GCAACAGGTTTTCACGGACT	O	O
Cbre_JC2_rev	AAGCACCCTGGTTTCATTCA	O	O
Cbre_JC3_For	AAAATTTGCACCTAATCCTGCT	O	O
Cbre_JC3_rev	GCATGGAAAAATTTATTCTGGA	O	O

BIONICS
